# Prediction of amyloid pathology in cognitively unimpaired individuals using voxel-wise analysis of longitudinal structural brain MRI

**DOI:** 10.1186/s13195-019-0526-8

**Published:** 2019-08-17

**Authors:** Paula M. Petrone, Adrià Casamitjana, Carles Falcon, Miquel Artigues, Grégory Operto, Raffaele Cacciaglia, José Luis Molinuevo, Verónica Vilaplana, Juan Domingo Gispert, for the Alzheimer’s Disease Neuroimaging Initiative

**Affiliations:** 1 Barcelonaβeta Brain Research Center (BBRC), Pasqual Maragall Foundation, C/ Wellington 30, 08005 Barcelona, Spain; 2 Department of Signal Theory and Communications, Universitat Politècnica de Catalunya, C/ Jordi Girona 1-3, edifici D5 Campus Nord UPC, 08034 Barcelona, Spain; 3 Centro de Investigación Biomédica en Red Bioingeniería, Biomateriales y Nanomedicina (CIBER-BBN), Madrid, 28029 Spain; 4 CIBER Fragilidad y Envejecimiento Saludable (CIBERFES), Madrid, Spain; 5 Universitat Pompeu Fabra, Barcelona, Spain

**Keywords:** Preclinical AD signature, Jacobian determinant, MRI, Machine learning, Longitudinal voxel-wise analysis

## Abstract

**Background:**

Magnetic resonance imaging (MRI) has unveiled specific alterations at different stages of Alzheimer’s disease (AD) pathophysiologic continuum constituting what has been established as “AD signature”. To what extent MRI can detect amyloid-related cerebral changes from structural MRI in cognitively unimpaired individuals is still an area open for exploration.

**Method:**

Longitudinal 3D-T1 MRI scans were acquired from a subset of the ADNI cohort comprising 403 subjects: 79 controls (Ctrls), 50 preclinical AD (PreAD), and 274 MCI and dementia due to AD (MCI/AD). Amyloid CSF was used as gold-standard measure with established cutoffs (< 192 pg/mL) to establish diagnostic categories. Cognitively unimpaired individuals were defined as Ctrls if were amyloid negative and PreAD otherwise. The MCI/AD group was amyloid positive. Only subjects with the same diagnostic category at baseline and follow-up visits were considered for the study. Longitudinal morphometric analysis was performed using SPM12 to calculate Jacobian determinant maps. Statistical analysis was carried out on these Jacobian maps to identify structural changes that were significantly different between diagnostic categories. A machine learning classifier was applied on Jacobian determinant maps to predict the presence of abnormal amyloid levels in cognitively unimpaired individuals. The performance of this classifier was evaluated using receiver operating characteristic curve analysis and as a function of the follow-up time between MRI scans. We applied a cost function to assess the benefit of using this classifier in the triaging of individuals in a clinical trial-recruitment setting.

**Results:**

The optimal follow-up time for classification of Ctrls vs PreAD was Δ*t* > 2.5 years, and hence, only subjects within this temporal span are used for evaluation (15 Ctrls, 10 PreAD). The longitudinal voxel-based classifier achieved an AUC = 0.87 (95%CI 0.72–0.97). The brain regions that showed the highest discriminative power to detect amyloid abnormalities were the medial, inferior, and lateral temporal lobes; precuneus; caudate heads; basal forebrain; and lateral ventricles.

**Conclusions:**

Our work supports that machine learning applied to longitudinal brain volumetric changes can be used to predict, with high precision, the presence of amyloid abnormalities in cognitively unimpaired subjects. Used as a triaging method to identify a fixed number of amyloid-positive individuals, this longitudinal voxel-wise classifier is expected to avoid 55% of unnecessary CSF and/or PET scans and reduce economic cost by 40%.

**Electronic supplementary material:**

The online version of this article (10.1186/s13195-019-0526-8) contains supplementary material, which is available to authorized users.

## Background

Despite enormous efforts, there is yet no disease-modifying treatment available for Alzheimer’s disease (AD). In this scenario, a promising strategy aims to prevent AD by developing interventions before the onset of symptoms [1]. The main challenge to operationalize such strategy lies in the detection of those individuals who are at increased risk to develop symptoms in the short term and would best benefit from these interventions [2].

Biomarker studies have demonstrated that AD pathology unfolds as a continuum [3]. AD starts with a dormant asymptomatic stage—the “preclinical state” (PreAD)—followed by the progressively impaired symptomatic states of mild cognitive impairment (MCI) and dementia. PreAD is characterized by unimpaired cognition, performance within norms taking into account age and education, and abnormal amyloid biomarkers as measured in cerebrospinal fluid (CSF) or by positron emission tomography (PET). The PreAD stage can last for decades and thus provides a window of opportunity for potential preventive intervention with disease-modifying therapies as long as the earliest pathophysiological changes that precede the emergence of AD clinical symptoms can be detected. However, CSF and PET are not suitable techniques for the screening or triaging of the general population given their invasiveness and high cost.

Recent developments in magnetic resonance imaging (MRI) permit the study of the neuroanatomy with unprecedented detail. MRI has proven to be instrumental at characterizing impending dementia and cognitive decline due to AD both for research and in the clinic [4]. The neuroimaging AD signature has been established as structural changes in AD-vulnerable structures (i.e., entorhinal cortex, hippocampus, and temporal lobe) that constitute diagnostic markers of cognitive impairment and AD progression [5, 6]. A *preclinical AD signature* might also be present in structural imaging, as several recent studies point out [7–10, 13], even though to a lower degree as what is observed in the clinical stages of the disease. On top of this, preliminary results by our group [13] and others [11] show that brain anatomical changes at the PreAD stage involve regions of the aforementioned AD signature.

In this line, artificial intelligence, hand in hand with MRI, come to the aid of early disease detection across a variety of medical domains. In the scope of AD, many efforts have been dedicated to the automated detection of mild cognitive impairment and dementia due to AD based on biomarkers and MRI-T1 images of subjects [12]. However, the detection of PreAD from MRI datasets has received much less attention. In a previous study based on brain regions of interest (ROIs), we showed that MRI in combination with machine learning can predict amyloid positivity with enough accuracy (AUC = 0.76) to be cost-effective as a pre-screening tool [13]. In that report, the MRI’s predictive capacity was validated in two independent cohorts and a similar cross-sectional study achieved similar results in a third population [14]. A good review of machine learning methods (feature extraction, feature selection, cross-validation, and classifier) using cross-sectional MRI can be found in [38]. In the present voxel-wise study, instead, we investigate how longitudinal brain structural changes in preAD and AD subjects differ from normal brain aging processes. Our longitudinal voxel-wise approach utilizes tensor-based morphometry to make inferences on local tissue gain or loss that occur over the different stages of AD. In tensor-based morphometry, a Jacobian determinant map is computed for the deformation field between a reference and a target image [39], or an average group template [40]. Hence, Jacobian determinant maps are interpreted as a measure of local tissue change, and previous studies show that this approach can achieve improved accuracy in the diagnostic classification of AD/MCI vs. controls [41, 42]. Our work is based on voxel-wise Jacobian determinant maps that capture structural changes in the brain between two points in time, and we focus on understanding how these changes differ between subjects at risk of AD and those subjects whose brain follow normal aging processes.

The objectives of this work are therefore twofold. On the one hand, we seek to identify the most significant features from the Jacobian determinant maps that can distinguish normal subjects from those with early asymptomatic AD stages. To achieve this aim, we implement a machine learning workflow with a cross-validation loop [24]. First, a voxel-wise feature selection step [43] distinguishes the most discriminant features in the Jacobian maps, and then, we use these features to predict amyloid positivity in early AD stages using a machine learning classifier. This novel classification model relies on longitudinal MRI images acquired throughout two time points and is able to predict amyloid positivity based solely on brain structural changes that are different to those that pertain to normal brain aging as shown in cognitively unimpaired and amyloid-negative individuals used as controls. We establish that a voxel-wise machine learning classifier based on Jacobian determinants provides higher accuracy than what was obtained using ROIs in our cross-sectional study, and therefore shows potential benefit as a screening tool in a clinical trial setting.

On a parallel and independent analysis, we seek to characterize the PreAD signature, as compared to that of AD. To achieve this aim, we carry out a statistical analysis of voxel-wise Jacobian determinant maps across the complete sample population and identify regions of stage-specific change with volume increase or decrease. At the voxel-wise level, we report a pattern of early brain structural changes that can be associated with disease progression and differ from normal aging and also to those observed in later AD stages.

## Methods

### Subjects

Subjects for this study were selected from the ADNI database [15] provided that they had two or more longitudinal 3D-T1 MRI acquisitions and cerebrospinal fluid (CSF) biomarker data publicly available. Subjects were assigned biomarker-assisted diagnostic categories following recently published guidelines [16]. Subjects labeled as “Normal” in ADNI were classified as amyloid negative cognitively unimpaired (Ctrl) if CSF Aβ was above 192 pg/mL and preclinical (PreAD) if CSF Aβ was below 192 pg/mL. This threshold has been shown to optimally discriminate between cognitively unimpaired individuals and AD patients and has been extensively used as cutoff value for amyloid positivity [17]. Subjects were categorized as MCI or AD according to the ADNI diagnostic categories reported in [18], and we selected only those individuals with CSF Aβ levels below 192 pg/mL to exclude subjects harboring non-AD pathological changes. At baseline, this diagnostic algorithm yielded 79 Ctrl, 50 PreAD, and 274 MCI/dementia due to AD, a total of 403 subjects with complete imaging and CSF data. As additional inclusion criteria, in follow-up visits, all subjects remain in the same diagnostic category. We exclude subjects that progress between diagnostic categories within the time span of the study due to small sample size (13 PreAD converters from Ctrls, 13 MCI/AD converters from PreAD, and 1 MCI/AD converter from Ctrl).

### MRI data

Structural 3D-T1 MRI images were acquired across different scanners and institutions. Each image was associated with a cognition score and a set of CSF biomarker values (amyloid-beta, total tau, and phosphorylated tau). The date of the CSF extraction was selected to be within 90 days from the date of the MRI scan. Each subject had at least one follow-up visit with the corresponding T1-MRI image, cognition score, and CSF biomarker values. The number of visits may differ across subjects (Table 1). The total number of MRI scans analyzed was 980. The time interval between visits was, at least, 6 months apart.

**Table 1:** Distribution of the number of 3D-T1 MRI acquisitions per subject

Number of visits	*N*
2	295
3	63
4	27
5	15
6	3
Total	403 subjects 841 Jacobian maps

### Image analysis

The SPM12 [19] neuroimaging software suite was used for every step of this longitudinal analysis pipeline. All image pairs corresponding to the same subject from the ADNI database were processed with longitudinal pairwise registration. Images in each pair were averaged and their respective Jacobian determinant was calculated, which reflects the regional cerebral volumetric changes between the respective time points. DARTEL normalization [31] was applied onto average images to normalize Jacobian determinant maps to MNI space [32] and allow comparison across subjects. The intensity of each voxel in the Jacobian image was normalized by the interval of time between reference and follow-up visits (i.e., Δ*t*). The number of Jacobian determinant maps for each subject’s diagnostic category is 184 Ctrl, 114 PreAD, and 543 MCI/AD.

On top of the voxel-wise analysis, a regional analysis was also performed. To this end, regions of interest (ROIs) in the AAL atlas were masked by each subject’s gray matter segmentation and the mean value of the remaining voxels’ intensity per region was computed [20].

### Automated recognition of PreAD volumetric changes using machine learning

All Jacobian determinant maps from each subject were labeled using subject’s label (i.e., PreAD, Ctrl), leaving a study cohort of *N* = 129 (*N*_Ctrl_ = 79, *N*_PreAD_ = 50). Importantly, as mentioned before, we only consider pairs of images for which no transitions have been observed across categories. This analysis was performed only on the PreAD and Ctrl subjects.

#### Feature selection

Due to the limited sample size and high dimensionality of the Jacobian determinant maps, we perform feature selection to keep an optimal percentage of the most relevant features. To this end, we use a filter feature selection method based on *F* test, taking into consideration Jacobian features and subject labels. The *F*-test metric is used to create a ranking of all Jacobian features and finally a fixed percentage of the highly ranked features are used for classification [22].

#### Classification and performance evaluation

**Fig. 1:**
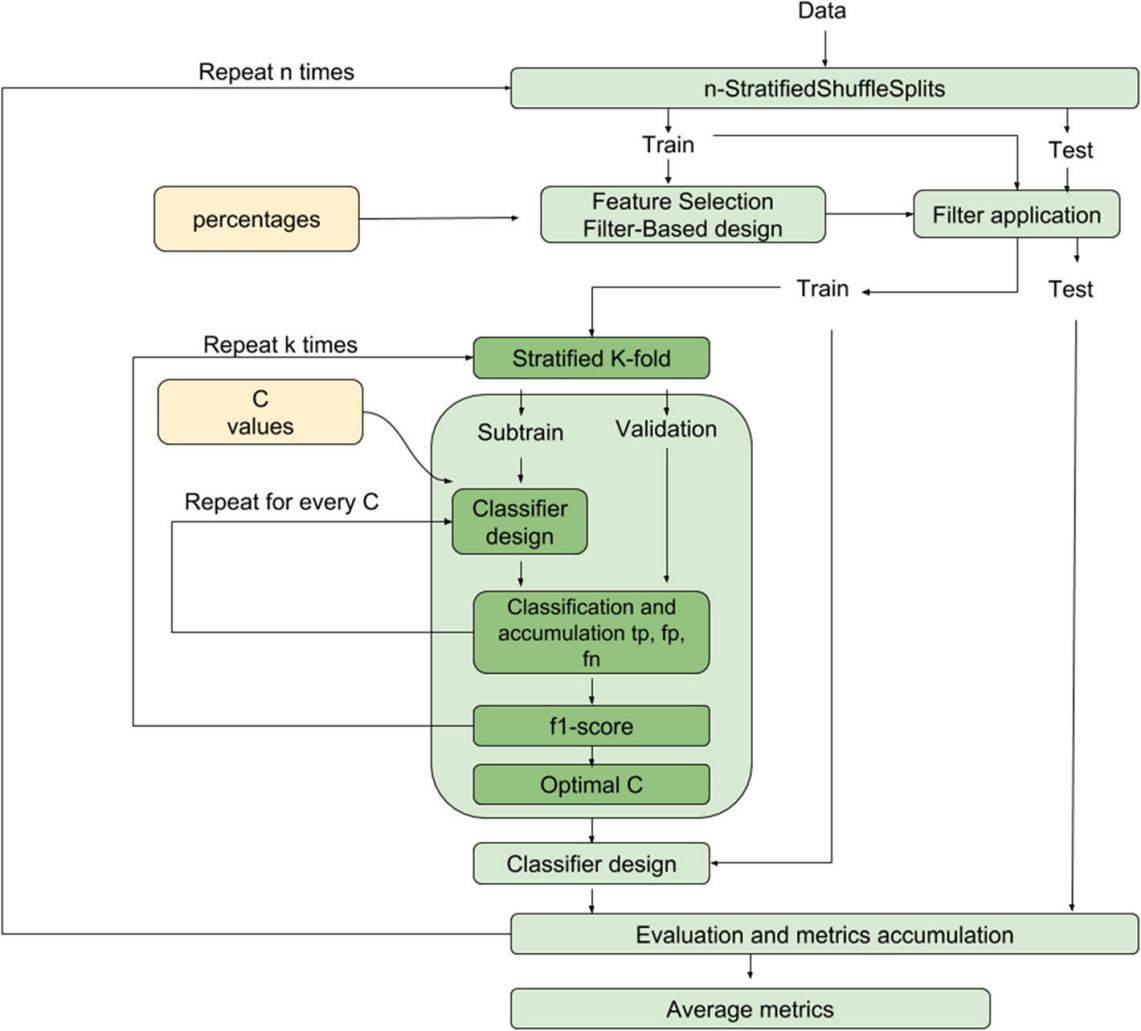
Workflow of the optimization and evaluation of the classification method. The performance of the final classifier is evaluated on a fresh test set that has not been used for training

Ridge logistic regression with hyperparameter C [23] is used for binary classification of Jacobian features within the nested cross-validation (CV) framework [24] defined in Fig. 1. It consists of an inner CV loop for model selection and an outer CV loop for assessing model performance. First, in the outer loop, subjects are randomly divided into 80% train set and 20% test set previously fixing a prevalence of interest (the percentage of samples of the amyloid positive class). For each subject in either set, all available Jacobian determinant maps are used for classification. The train set is used for feature selection and model optimization while the test set is left out for final model evaluation. The random split by subject ensures that there is no contamination of the test set with Jacobian determinants of the train set.

Feature selection is computed using only the train set. In the model optimization step, the train set is further split into sub-train (2/3) and validation (1/3) sets using a (*k* = 3)-fold cross-validation. A grid search strategy is used to optimize the classifier hyperparameter C by maximizing the f1-score on the validation set. Finally, the model is estimated using the optimized hyperparameter C on the whole train set. Then, the model is applied to the test set to compute standard performance metrics (i.e., area under the receiver operating curve (AUC), accuracy, precision, sensitivity, specificity, and f1-score). Following the formulation in [13], we also report the reduction of economic cost (i.e., savings) of using this classification framework as a tool for AD screening.

This procedure is repeated *n* = 100 times, and performance results are reported using the average and standard deviation. The overall implementation is based on the scikit-learn Python library (version 0.18) [25].

#### Savings

Savings in a triaging process were calculated as the percentage difference in resources between the standard recruitment protocol and using our proposed protocol in [13] to obtain a desired number of PreAD subjects for the clinical study. Savings were assessed in terms of economic cost (Eq. 1) or participant burden (Eq. 2), i.e., the amount of unnecessary PET/CSF tests spared through MRI screening.

1 $$ \mathrm{Saving}{\mathrm{s}}_{\mathrm{COST}}=1-\frac{1}{2\cdot {C}_{\mathrm{avg}}}\left(\rho \frac{C_{\mathrm{PET}}}{P}+\frac{C_{\mathrm{MRI}}}{R}\right) $$

2 $$ \mathrm{Saving}{\mathrm{s}}_{\mathrm{CSF}/\mathrm{PET}}=1-\rho\ \frac{1}{P} $$

Savings rely on the algorithmic precision (*P*) and recall/sensitivity (*R*) and on the prevalence of the population (*ρ*). The costs of MRI and PET were estimated as *C*_MRI_ = 700 € and *C*_CSF_ = 3000 €, and *C*_avg_ represents the average cost among the screening tests which may include additional costs (e.g., neuropsychological cognitive testing).

#### Statistical analysis

The aim of the statistical analysis is to identify significant group differences in brain volumetric change rate between AD stages. We will investigate the location of these stage-specific changes and whether they represent a volume increase (positive changes) or decrease (negative changes). Every Jacobian determinant map is treated as an independent variable.

##### Two-sample *t* test

Statistical analyses were performed by comparing any combination of two subject categories. The uncorrected threshold for statistical significance was *p* < 0.005. Spatial clustering of regions with statistically relevant voxels was applied to rule out false-positives, with a clustering threshold of *k* > 100 voxels under which voxel clusters with smaller sizes were discarded.

##### Data normalization

The effects of normal aging on brain structural changes were considered as a confounder and regressed out [21]. Coefficients for linear regression on age were fitted using only Ctrls (i.e., individuals that are amyloid negative, asymptomatic in all visits).

The age corresponding to each Jacobian determinant was defined as the mean age between the two visits, i.e., age *=* (age_reference_ + age_follow-up_)/*2*.

## Results

### Demographic and follow-up comparisons

We included a total of 403 subjects at baseline with at least one follow-up visit over three categories: Ctrl (*n* = 79), PreAD (*n* = 50), and MCI/AD (*n* = 274). Demographic data and follow-up period are presented in Table 2 split into different categories.

**Table 2:** Dataset demographics at baseline

Category	Ctrl (Aβ-)	PreAD (Aβ+)	MCI (Aβ+)	AD (Aβ+)	MCI/AD (Aβ+)
Number of subjects	79	50	196	78	274
Age (years) at baseline (mean; std)	73.97 (5.97)	76.04 (6.25)	73.55 (6.55)	75.44 (7.37)	74.1 (6.85)
Sex (F/M)	37/42	21/29	79/117	33/45	112/162
Follow-up (years) period (mean; std)	2.48 (1.38)	2.32 (1.32)	2.2 (1.09)	1.4 (0.46)	1.97 (1.02)

We denote as Δ*t* the time interval between two follow-up visits (i.e., reference and target images).

**Fig. 2:**
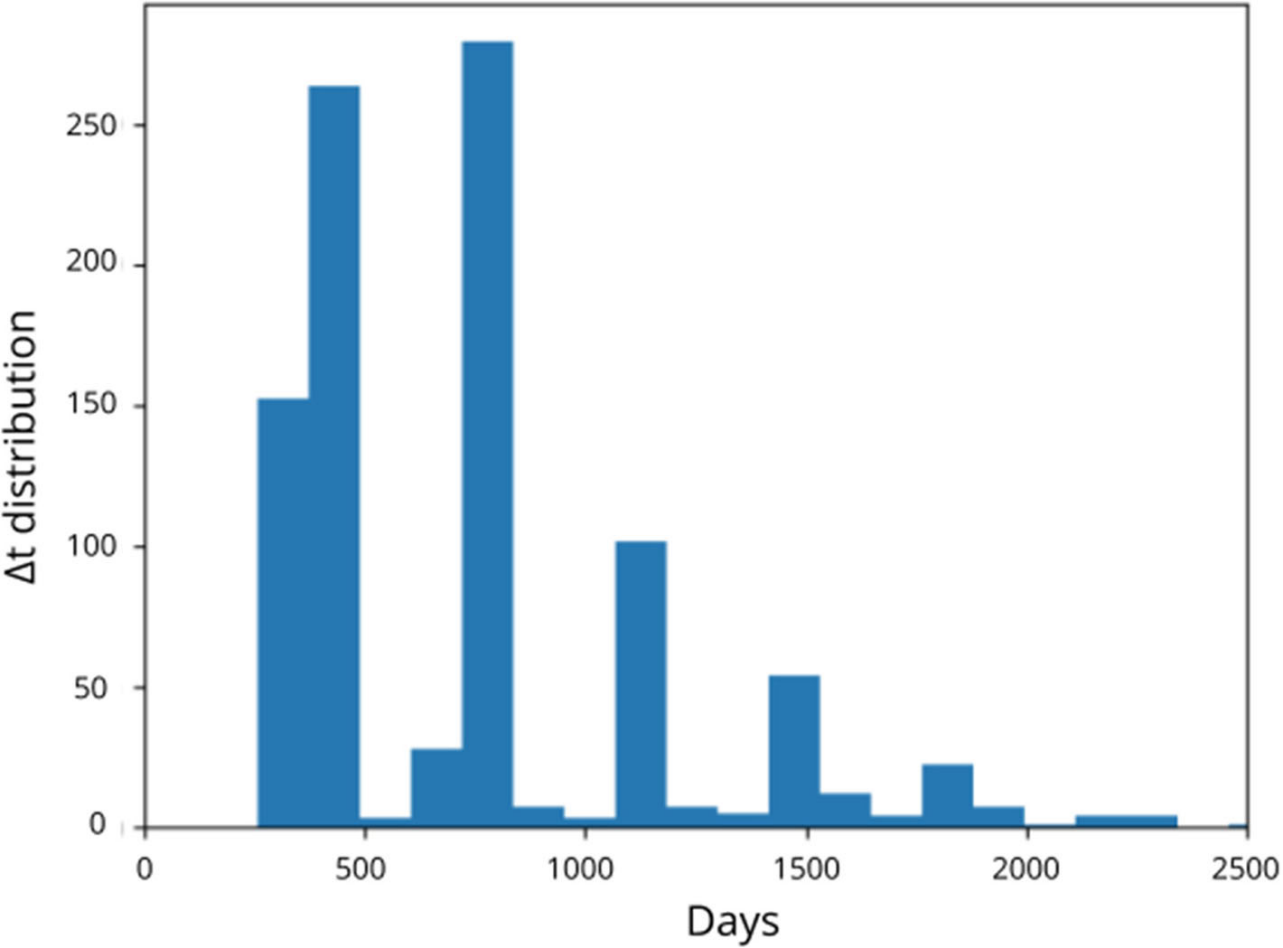
Distribution of the interval Δ*t* between reference and follow-up visits across the whole dataset

The distribution of the time interval (Δ*t*) between follow-up visits on all subjects is given in Fig. 2. The median of the distribution is 2.01 years*.*

A subset of the cohort for which 3.5 > Δt > 2.5 years was used for some of the machine learning studies, given that longer Δ*t* account for more signal-to-noise ratio into disease progression. Demographics for this subset of subjects are provided in Table 3. Additional file 2: Table S2 provides information about the research facility and type of scanner that were used for each of the subjects in this reduced cohort.

**Table 3:** Demographics of the subset of the study cohort for which Δ*t* > 2.5 used for machine learning classification

Category	Ctrl	PreAD	MCI	AD	MCI/AD
Number of subjects	15	10	38	0	38
Age (years) at baseline (mean; std)	76.51 (6.18)	76.0 (3.97)	72.987 (6.11)	–	72.987 (± 6.11)
Sex (F/M)	8/7	5/5	27/11	–	27/11
Follow-up (years) period (mean; std)	4.17 (1.035)	4.21 (0.98)	3.91 (0.82)	–	3.91 (0.82)

### Machine learning

We use machine learning for voxel-wise prediction of amyloid-positive subjects (PreAD) among cognitively unimpaired subjects. A realistic prevalence for PreAD subjects on middle-age adults is 20% [26]. We use this prevalence to fix the proportion of PreAD on the test set on all machine learning experiments, including the training of the classifier.

**Fig. 3:**
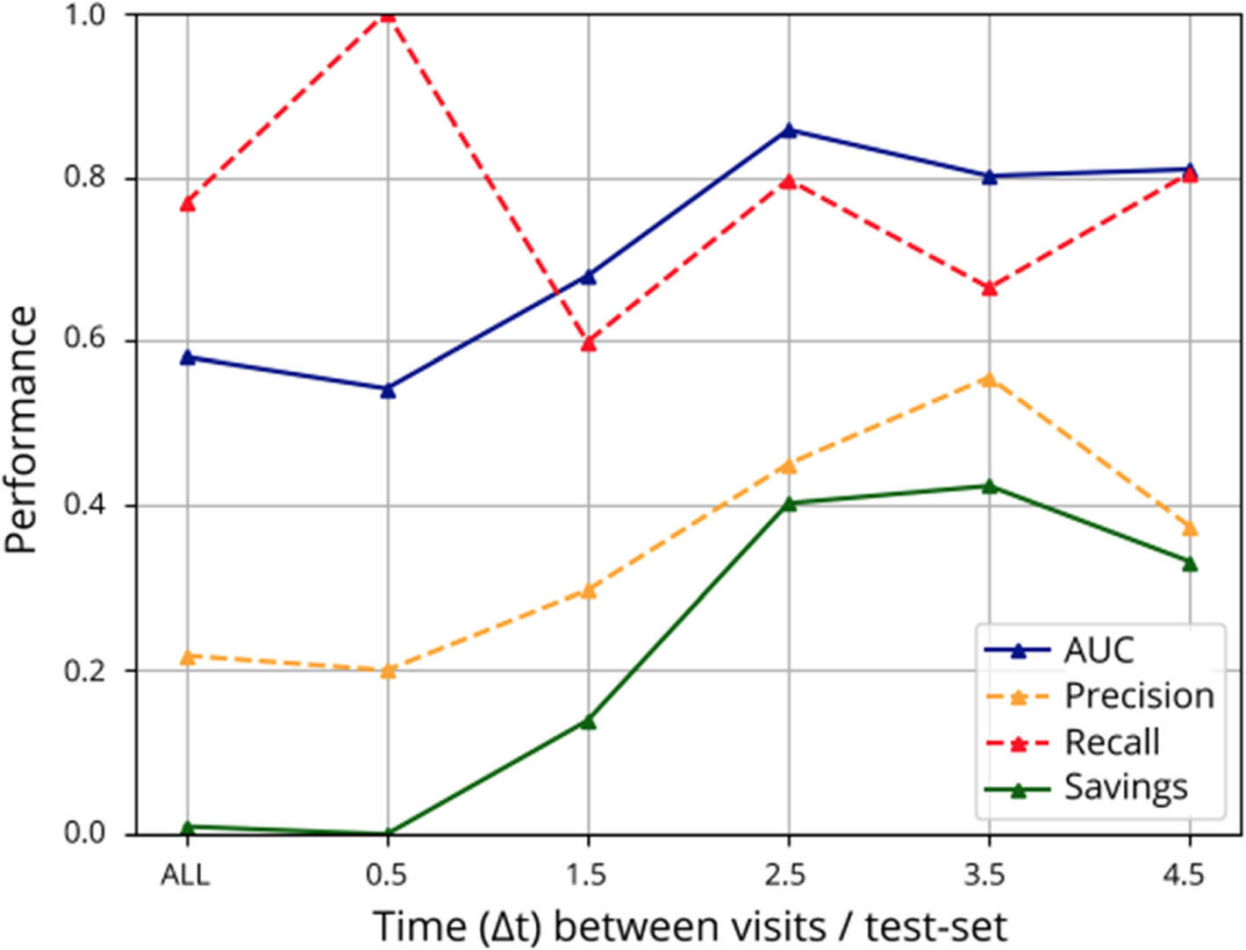
AUC and savings (blue, green) reported using Jacobian determinant maps with different time intervals (Δ*t*) between reference and target and a fixed prevalence of 20% amyloid-positive subjects on the test set. To compute savings, we used optimal precision and recall values plotted in dashed orange and red lines, respectively using the cost function defined in Eq. 1

Another key parameter of the analysis is the temporal distance (Δt) between reference and target images used to compute the Jacobian determinant maps. In Fig. 3, we report the performance of the classifier as a function of minimal Δ*t* values in the test set. It is observed that even though we normalize each Jacobian determinant map with respect to the Δ*t* parameter, the preclinical signature is within the detection range when visits are at least 2.5 years apart. In the case in which Δ*t* > 2.5 years, the performance of the classifier based on structural changes is much better than a classifier trained on individual images as reported in our previous cross-sectional study that reports an AUC = 0.76 [13]. When using Jacobian determinant maps with smaller temporal distance (Δ*t* < 2.5 years), the mean performance is worse than the cross-sectional analysis, probably due to the low signal-to-noise ratio between the changes due to normal brain aging and the changes due to amyloid positivity [13].

The optimal temporal span in terms of AUC and savings between data acquisitions is Δ*t* > 2.5 years. The number of subjects with follow-up visits between 2.5 < Δ*t* < 3.5 years from baseline is reduced to 15 Ctrls, 10 PreAD, and 38 MCI/AD subjects with 25, 16, and 52 Jacobian determinant maps, respectively. In what follows, throughout the paper, we use only Jacobian determinant maps within the optimal temporal span (2.5 < Δ*t* < 3.5 years) for evaluation purposes. The use of Jacobians within this temporal span (2.5 < Δ*t* < 3.5 years) for training the system and evaluating it in all other cases has also been tested, with poor generalization (Additional file 4: Table S4).

**Fig. 4:**
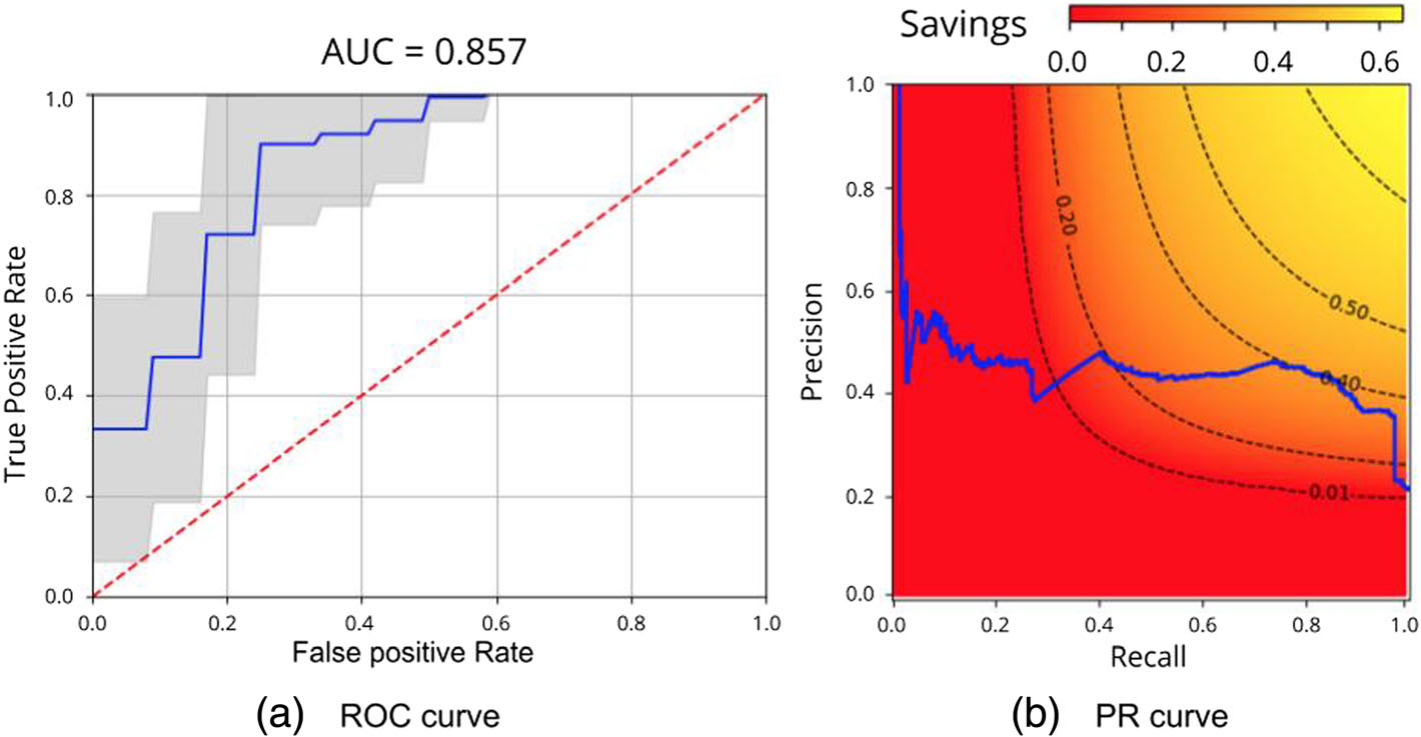
ROC and PR curves for Jacobian determinant maps with time spans in the range 2.5 < Δ*t* < 3.5 years using 0.5% of the features. On the left, the ROC curve is averaged across different development/test splits: the mean curve (blue) with the standard deviation (gray) and the curve of a random classifier (red). On the right, the PR curve of the classifier (blue) is overlaid on a savings heatmap (Eq. 1). Black lines indicate points of equal savings

Receiver operating characteristic curve (ROC) and precision-recall (PR) curves of the classifier are shown in Fig. 4. A savings heatmap that responds to Eq. 1 is overlaid on the PR curve, while the mean and standard deviation of the model performance are plotted against the random classifier on the ROC curve.

The impact of different number of features used to train our multivariate algorithm is presented in Table 4, evaluated on our dataset which is imbalanced (36% of preclinical subjects). Note that the prevalence of preclinical subjects on the test set is forced to 20% using permutations. When using a low number of features, the model underrepresents the preclinical signature, not being able to capture all data heterogeneity. In contrast, when using a high number of features, the model is not able to generalize results to unseen Jacobian determinant maps, overfitting the development set. Hence, the best results are obtained using a moderate number of features that are able to both represent the preclinical signature and still generalize well to the test set. We also tested an embedded, multivariate feature selection method based on l1-norm minimization resulting in lower performance (Additional file 3: Table S3).

**Table 4:** Performance of the system using a different number of features evaluated on the interval 3.5 > Δ*t* > 2.5 years

# features (%)	AUC (95% CI)	Balanced accuracy (95% CI)	Accuracy (95% CI)	Precision (95% CI)	Sensitivity (95% CI)	Specificity (95% CI)	F score (95% CI)
6 (0.00)	0.78 (0.50–0.99)	0.70 (0.375–0.875)	0.57 (0.20–0.80)	0.33 (0.13–0.50)	0.91 (0.33–1.00)	0.48 (0–0.75)	0.48 (0.19–0.67)
65 (0.01)	0.81 (0.60–0.97)	0.74 (0.478–0.834)	0.63 (0.27–0.73)	0.38 (0.18–0.43)	0.91 (0.33–1.00)	0.56 (0.12–0.75)	0.50 (0.28–0.6)
653 (0.10)	0.85 (0.67–1.0)	0.77 (0.60–0.88)	0.65 (0.53–0.8)	0.37 (0.27–0.50)	0.97 (0.67–1.00)	0.57 (0.42–0.75)	0.53 (0.38–0.67)
1633 (0.25)	0.86 (0.72–1.00)	**0.77 (0.67–0.88)**	0.65 (0.46–0.8)	**0.37 (0.27–0.50)**	**0.98 (0.67–1.00)**	0.53 (0.33–0.75)	**0.53 (0.43–0.67)**
3266 (0.50)	0.86 (0.71–0.97)	0.77 (0.60–0.88)	0.64 (0.46–0.8)	0.36 (0.26–0.50)	0.97 (0.67–1.00)	0.56 (0.33–0.75)	0.53 (0.38–0.67)
6532 (1.00)	**0.87 (0.72–0.97)**	**0.76 (0.58–0.88)**	**0.65 (0.53–0.80)**	0.36 (0.25–0.50)	0.933 (0.67–1.00)	**0.58 (0.42–0.75)**	0.52 (0.36–0.67)
13,064 (2.00)	0.86 (0.64–1.00)	0.75 (0.50–0.88)	0.65 (0.46–0.80)	0.36 (0.20–0.50)	0.917 (0.67–1.00)	0.58 (0.33–0.75)	0.51 (0.31–0.67)
32,661 (5.00)	0.80 (0.49–1.00)	0.67 (0.42–0.86)	0.57 (0.40–0.77)	0.30 (0.14–0.47)	0.837 (0.33–1.00)	0.50 (0.33–0.71)	0.44 (0.33–0.71)
65,323 (10.00)	0.77 (0.40–1.00)	0.66 (0.42–0.86)	0.573 (0.4–0.77)	0.298 (0.14–0.47)	0.813 (0.33–1.00)	0.51 (0.33–0.75)	0.43 (0.33–0.75)

Figures in bold represent the maximum for each performance criterion

**Fig. 5:**
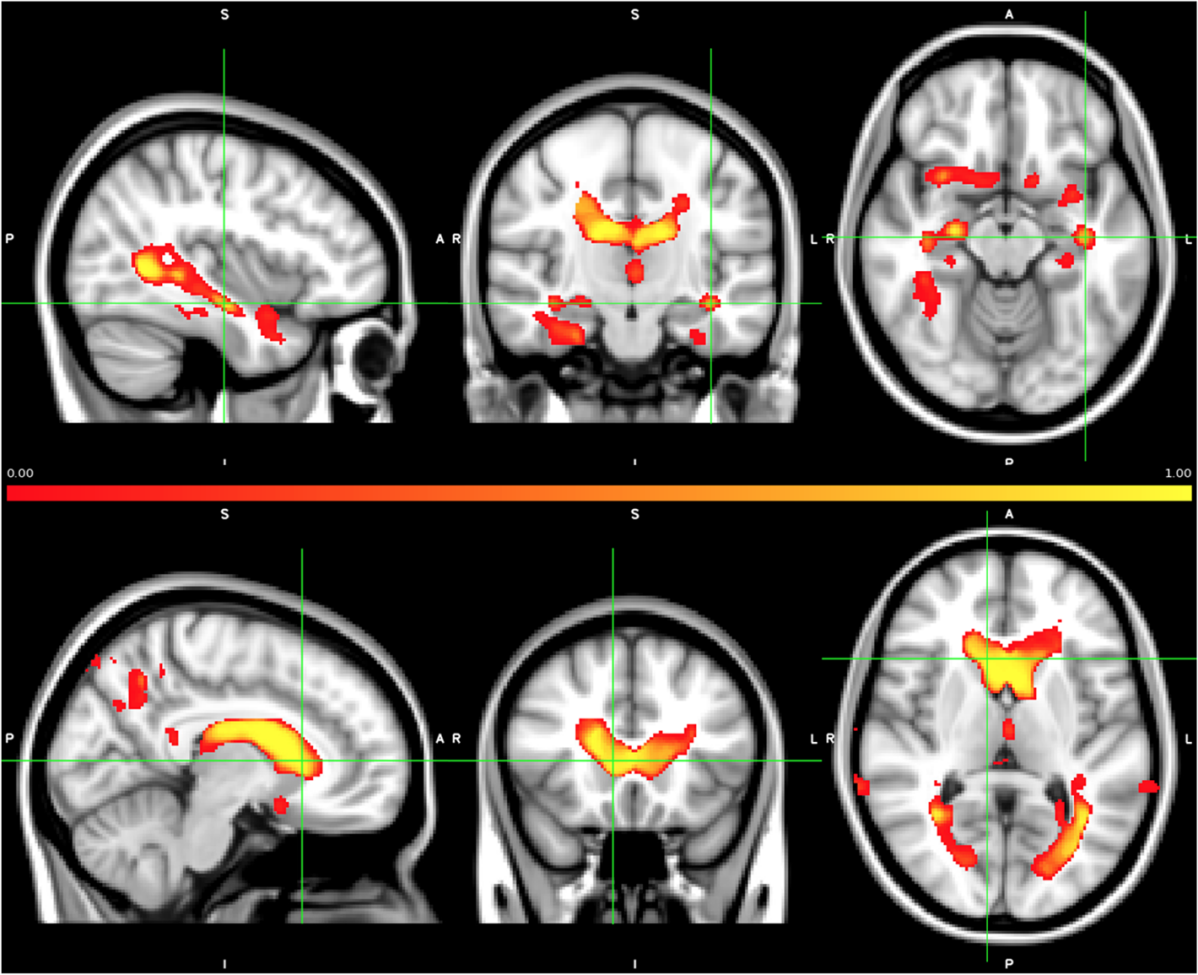
Normalized feature maps of the 0.5% of features selected during the 100 different splits of the development/test sets, representing the frequency of selection of each feature. Those features have optimal capacity to detect the presence of early amyloid pathology in asymptomatic individuals

An optimal compromised solution between several metrics is to design our model using 0.5% of the total Jacobian features. In this case, after the 100 iterations of the nested cross-validation framework, a heatmap of selected features is shown in Fig. 5. As expected, the top selected features correspond to typical regions affected by AD pathology like the caudates, fusiform, or parahippocampal gyrus, presenting high overlap with the statistical analysis presented in the next section. This result shows that a machine learning classifier trained on changes in specific brain regions has the capacity to predict the presence of early amyloid pathology in asymptomatic individuals as measured by MRI.

### Preclinical AD volumetric changes

**Fig. 6:**
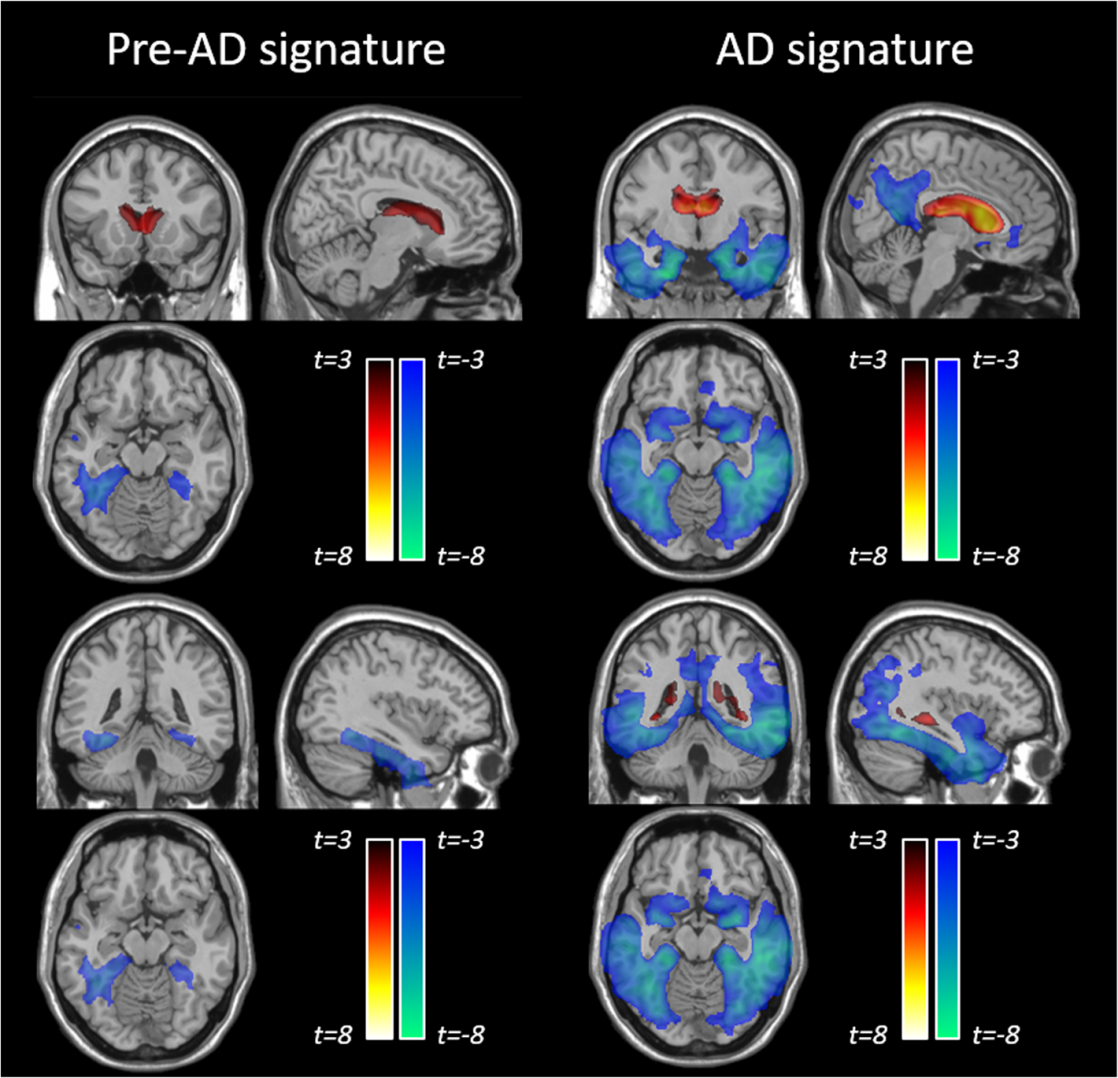
Statistical maps for group comparison between Ctrl and PreAD (PreAD signature) and Ctrl and MCI/AD (AD signature) subjects. Statistical significance was set to uncorrected *p* value < 0.005 and minimum spatial extent *k* > 100

In parallel to the machine learning classification model, we carried out a voxel-wise statistical analysis using the full dataset of Jacobian determinant maps to identify the regions of volumetric change that are statistically significant between the different categories Ctrl, PreAD, and AD/MCI (Fig. 6).

Stable PreAD individuals show significantly higher gray-matter (GM) atrophy in the parahippocampal and fusiform gyri as compared to amyloid-negative cognitively unimpaired subjects, as shown on the left hand side of Fig. 6. Apparent mild GM increments are detected in the caudate heads, probably as a surrogate effect of ventricular expansion.

Furthermore, comparison of longitudinal volumetric changes between amyloid-negative cognitively unimpaired subjects and stable symptomatic ones (amyloid positive MCI or AD subjects) reveals the well-known AD signature involving temporo-parietal and posterior cingulate areas, as well as most of the basal ganglia [27], as shown on the right hand side of Fig. 6. Of note, apparent GM increments are also detected in periventricular areas, including the caudates and medial thalamus.

## Discussion

The goal of this work was to assess whether brain structural changes captured by subsequent magnetic resonance images can indicate the presence of abnormal amyloid levels in cognitively unimpaired subjects using machine learning techniques. In addition, we also aimed at characterizing the preclinical signature voxel-wise using Jacobian determinant maps as a measure of volumetric rate of change.

A machine learning framework was implemented for the classification of amyloid-positive subjects using Jacobian determinant maps as features for classification. The best achieved performance in our longitudinal classifier (AUC 0.87) significantly improved the performance we previously reported for a cross-sectional classifier (AUC 0.76) [13]. This performance is significantly higher than what was reported in previous works that, on top of using MRI ROI data, built classifiers adding demographics (AUC 0.63), demographics and genetics (AUC 0.62–0.66), and demographics, neuropsychology, and APOE (AUC 0.74) [14, 28]. It is possible that adding complementary information to the MRI such as demographics and genetic risk factors may improve the performance of our machine learning classifier. While the field strength of the scanners is 1.5 T for all subjects, there is large heterogeneity in the site ID, so we believe this has had small or no influence on the performance metrics of the classifier.

The increased performance of our classifier may be accounted for two factors. On the one hand and unlike similar previously reported classifiers, we used voxel-wise data as features. Coupled with an efficient feature selection strategy, this allowed the classifier to select the most discriminant brain regions, independent of a priori cortical parcellations. On the other hand, we used subsequent images that correspond to the same individuals, thus eliminating an important percentage of the between-subject variability present in cross-sectional setups.

In this regard, we observed that our classifier works significantly better only when the pairs of MRI scans that are used for evaluation are acquired more than 2.5 years apart. This time period is likely related to the protracted evolution of the neuroanatomical changes in preclinical AD stages. At more advanced stages of the disease, more rapid evolution of brain structural changes is expected, and thus, the benefits of a longitudinal classifier would potentially be evident with shorter time intervals. It remains to be explored how these promising results would be affected by the use of different scanners. Still, a time gap of 2.5 for resolving preAD is within the timescale of relevance for AD screening or the follow-up of subjects enrolled in secondary prevention clinical trials, which typically last a decade. In this context, this work and our earlier study on MRI using ML [13] show that even though the performance of the ML classifier is not high, if implemented as a screening tool it can save resources in a clinical trial setting.

The main discriminative features between amyloid positive and healthy controls mostly included AD-related areas in the medial and inferior temporal lobe, as well as the lateral ventricles which can be considered as the *preclinical AD signature*. Increased expansion of the lateral and inferior lateral ventricles in cognitively unimpaired individuals with lower levels of CSF amyloid-beta has been shown previously, along with increased atrophy in the fusiform gyri as well as in middle temporal and posterior cingulate cortices [33–37]. In this regard, the preclinical AD signature found in our study does not significantly depart from published reports and, as can be seen in Fig. 6, is very much in line with the expected pattern of atrophy in AD, though to a lesser magnitude and extent.

In addition to (peri)ventricular regions, Fig. 5 also shows the fusiform gyri and middle temporal regions to display significant discriminative capacity to discriminate amyloid-positive vs amyloid-negative CU individuals, as expected [34]. Additional detail on the brain areas contributing to such discriminative power is now provided in Additional file 1: Table S1.

The predictive capacity achieved by this classifier does not place this method as substitute of gold-standard tests to detect amyloid abnormalities. Still, if used for triaging of subjects, e.g., clinical trial recruitment, we demonstrated that it could allow significant savings in terms of the number of costly gold-standard tests that would have to be performed to detect a fixed number of amyloid-positive, cognitively healthy subjects. Used in this way, in a cognitively unimpaired population with a prevalence of amyloid positivity of 20%, the accuracy of the longitudinal classifier would allow a reduction of up to 55% of unnecessary PET or CSF tests, which translates to a 40% reduction of the total cost, according to the savings model we previously proposed [13]. Nevertheless, in a clinical trial recruitment setting, it can be more advantageous instead to optimize the sensitivity of the classifier to maximize the number of detected at-risk individuals, at the cost of a slightly poorer specificity which might decrease these cost savings.

Due to the limited sample size for training and the large inter-subject variability of cerebral morphology, we use a simple but effective model for prediction of amyloid positivity. Our method is fully automatic from feature extraction and signature learning to classification. However, the presence of high-dimensional and low informative features together with the overlap between normal aging and AD processes in the brain reduces the overall precision of the system. To account for that, future efforts will need larger longitudinal datasets and many initiatives are contributing to achieve this [14, 29].

We observe much higher sensitivity than specificity. This is likely given the limited size and imbalance of the cohort but also most likely due to the fact that we are imposing an imbalance on the test set to simulate the preAD prevalence of 20% typically found in a clinical trial setting.

On top of this, given the limited sample size and the large amount of features used for classification (voxels), we might have incurred in an overfitting of the existing data, potentially resulting in an overestimation of the capacity of the classifier. Therefore, our results need to be validated on independent datasets, but the scarcity of longitudinal MRI datasets with CSF biomarker levels has prevented us to conduct such validation in this work. Still, in our previous ROI-based study, we successfully validated a very similar classifier with two independent datasets without a major loss of the classifier’s performance [13].

To further characterize the *preclinical AD signature*, a statistical analysis was conducted and we report longitudinal morphological changes in cognitively unimpaired subjects with abnormal amyloid CSF levels. This preclinical AD signature comprises atrophy of the parahippocampal and fusiform gyri and expansion of the lateral ventricles. This pattern is in line with previous reports of longitudinal volumetric changes associated with the presence of abnormal amyloid levels from ADNI participants that have been replicated in an independent cohort [10]. On the other hand, expansion of the caudate heads falls beyond this known pattern. Being in the proximity of the lateral ventricles, it may be questioned whether the detected increase in the volume of the caudates is an actual feature associated to preclinical AD stages or an artifact of the processing methodology to detect volumetric changes. By smoothing spatially continuous Jacobian determinant maps, it could be considered that the observed increase in caudate volumes could be a side effect of the “spillover” of the Jacobian determinant maps due to the expansion of the ventricles. To address this question, we performed a post hoc analysis of the caudate volumes between the Ctrls and PreAD groups, but using the longitudinal Freesurfer pipeline to compute change in caudate volumes. Since the subcortical segmentation implemented in Freesurfer uses an ROI approach based on a probabilistic atlas [30], it can be considered to be virtually free from the potential spillover effect of continuous Jacobian determinant maps. Results show that the changes in caudate volumes are not significantly different between Ctrls and PreAD individuals (*p* > 0.3) and, thus, it can be concluded that the observed caudate head expansion is artifactual and secondary to ventricular expansion. Still, this signal might contribute to the detection of the presence of amyloid burden in cognitively unimpaired individuals.

This study has some limitations. Even though data comes from a heterogeneous sample with different sites, and MRI scanners, the MRI acquisition was harmonized according to the ADNI protocol. Therefore, the performance of our method when applied to MRI samples using different acquisition protocols may deviate from what is here reported. Actually, the ultimate validation of the generalizability of the results here reported can only be accomplished by applying the method here developed to an independent sample. In our previous work, the performance of a similar cross-sectional classifier was kept stable when derived and validated in two independent cohorts. Therefore, it can be expected the same behavior in this longitudinal extension of the classifier. Our study relies on the ADNI cohort which is well-known for its data quality and unique in having corresponding MRI and CSF data and a longitudinal aspect required for a study using Jacobian determinants. The low amount of subjects with MRIs acquired with more than 2.5 years needed for a good signal to noise ratio certainly impose a limitation to our results and encourage future validation efforts. For example, one misclassification error has a huge impact on the performance metrics. To mitigate this effect, we repeated the workflow 100 times in order to report mean performance metrics. Nevertheless, the effect of misclassification can still be observed in the large confidence intervals that are found for each one of the metrics.

Finally, we used CSF amyloid as the gold-standard for amyloid positivity and not PET imaging. It could be argued that the performance of the classifier could be sensitive to the selection of the gold-standard method. However, the agreement between CSF and PET determinations of amyloid is very high, particularly in the intermediate ranges where thresholds for positivity typically lie.

One interesting area for further exploration is the classification subjects that undergo a transition between normal and preclinical amyloid biomarkers within the timeframe of two consecutive scans. In principle, one could hypothesize that this category of “transitioning” subjects will not necessarily follow the same pattern of brain volumetric change as either the normal or the preclinical group.

Unfortunately, only a subset of 13 subjects respond to these criteria; from these, only 2 subjects undergo this transition within a time frame of dt < 2.5 years between consecutive scans. The sample size is therefore too small for a machine learning workflow. Nevertheless, the prediction of a transition from normal to preclinical AD stages is a question of utmost importance to research (e.g., observational studies) and clinical practice (e.g., clinical trials) and a natural follow-up to the present study.

To sum up, we here presented a machine learning framework used to predict the presence of amyloid abnormalities in cognitively unimpaired individuals with a moderate-to-high accuracy (AUC 0.87) when MRI scans acquired 2.5 years apart are available. This performance translates to improvements of up to 55% in the number of necessary CSF/PET tests and a reduction of 40% of the costs to detect a fixed number of amyloid-positive individuals. This performance may still have room for improvement by including demographic, genetic, and cognitive data to the classifier. We further compare the features used by the classifier with the characteristic pattern of longitudinal morphological changes in preclinical AD that is expressed in typical AD-related regions, uncovering areas that appear to be specific to the preclinical AD stage.

## Conclusions

In this study, we used longitudinal structural brain MRI scans to predict the presence of amyloid pathology in cognitively unimpaired individuals and unveil the preclinical AD signature. We applied machine learning techniques on Jacobian determinant maps coding longitudinal volumetric changes at the voxel level. This allowed the classifier to significantly improve its performance (AUC = 0.87) with respect to previous cross-sectional ROI-based approximations. Areas showing the most discriminant capacity included medial, inferior, and lateral temporal regions, along with the ventricles and caudate heads. The volumetric changes in these areas are in line with those observed in symptomatic stages, but are expressed to a lower extent. Even though the performance of the classifier does not allow for it to substitute gold-standard methods to determine the presence of amyloid pathology, its use as triaging tool would lead to significant reductions of 55% of unnecessary gold-standard tests and of 40% of the cost to detect a fixed number of cognitively healthy individuals in preclinical AD stages. High overlap by the features used by the classifier and the preclinical AD signature is found, characterized by parahippocampal and fusiform gyri atrophy and expansion of the ventricles. To sum up, machine learning over brain longitudinal MRI data can represent a valuable tool for the implementation of secondary prevention trials. Statistical analysis of this longitudinal MRI data identified patterns of longitudinal brain structural changes specific to preclinical AD, as compared to those in MCI/AD subjects.

## Additional files

Additional file 1: **Table S1.** Percentage of discriminant voxels that correspond to each of the brain regions of interest (ROIs). (DOCX 23 kb)

Additional file 2: **Table S2.** List of cognitively unimpaired subjects with follow-up times in the interval 3.5 > Δt > 2.5 years used in the analysis. We report their ADNI ID, the research facility and type of scanner. (DOCX 90 kb)

Additional file 3: **Table S3.** Performance of the system using a different feature selection method (l1-norm selection) and evaluated on the interval 3.5 > Δ*t* > 2.5 years. The number of features depends on the l1-norm regularization parameter and on the training data. We report the average number of features used periteration of the evaluation loop. (DOCX 14 kb)

Additional file 4: **Table S4.** Performance of the system trained on the interval 3.5 > Δ*t* > 2.5 years and evaluated in all other cases. (DOCX 15 kb)

## Data Availability

Data used in the preparation of this article were obtained from the ADNI database (adni.loni.usc.edu), which is easily available for download from the Laboratory of Neuroimaging (LONI) website to the research public.

## Abbreviations

AD: Alzheimer’s disease
AUC: Area under the curve CV Cross-validation
CI: Confidence interval
CSF: Cerebrospinal fluid
Ctrls: Control subjects
GM: Gray-matter
MCI: Mild cognitive impairment
MNI: Montreal Neurological Institute
MRI: Magnetic resonance imaging
PET: Positron emission tomography Aβ Amyloid-beta
PreAD: Preclinical Alzheimer’s disease
ROI: Region of interest
SPM: Statistical parametric mapping
