# Supplementary material for: Prediction of amyloid pathology in cognitively unimpaired individuals using voxel-wise analysis of longitudinal structural brain MRI
Source: Alzheimers Res Ther. 2019 Aug 17;11:72. doi: 10.1186/s13195-019-0526-8 (PMC6698344; doi:10.1186/s13195-019-0526-8)
Supplement: Supplementary file 1 — Table S1. Percentage of discriminant voxels that correspond to each of the brain regions of interest (ROIs). (DOCX 23 kb) [file 13195_2019_526_MOESM1_ESM.docx]

| **Table S1. Percentage of discriminant voxels that correspond to each of the brain regions of interest (ROIs)** | | | |
| --- | --- | --- | --- |
|  |  |  |  |
| **ROI_Number** | **ROI_Description** | **Percentage_of_relevant voxels** | **Minimum_relevant_voxels_per_iteration** |
| 1 | Left Precentral | 0 | 0 |
| 2 | Right Precentral | 3,19E-03 | 7,65E-05 |
| 3 | Left Superior Frontal | 0 | 0 |
| 4 | Right Superior Frontal | 0 | 0 |
| 5 | Left Superior Frontal Orbital | 0 | 0 |
| 6 | Right Superior Frontal Orbital | 9,33E-04 | 3,26E-05 |
| 7 | Left Middle Frontal | 0 | 0 |
| 8 | Right Middle Frontal | 0 | 0 |
| 9 | Left Middle Frontal Orbital | 0 | 0 |
| 10 | Right Middle Frontal Orbital | 0 | 0 |
| 11 | Left Inferior Frontal Operculum | 0 | 0 |
| 12 | Right Inferior Frontal Operculum | 2,96E-03 | 1,15E-04 |
| 13 | Left Inferior Frontal | 0 | 0 |
| 14 | Right Inferior Frontal | 0 | 0 |
| 15 | Left Inferior Frontal Orbital | 0 | 0 |
| 16 | Right Inferior Frontal Orbital | 0 | 0 |
| 17 | Left Rolandic Operculum | 0 | 0 |
| 18 | Right Rolandic Operculum | 3,97E-03 | 7,28E-05 |
| 19 | Left Superior Motor | 0 | 0 |
| 20 | Right Superior Motor | 0 | 0 |
| 21 | Left Olfactory | **1,23E-01** | 8,82E-03 |
| 22 | Right Olfactory | **2,04E-01** | 9,78E-03 |
| 23 | Left Superior Medial Frontal | 0 | 0 |
| 24 | Right Superior Medial Frontal | 0 | 0 |
| 25 | Left Medial Frontal Orbital | 0 | 0 |
| 26 | Right Medial Frontal Orbital | 0 | 0 |
| 27 | Left Rectus | 1,37E-02 | 4,42E-04 |
| 28 | Right Rectus | 3,40E-02 | 1,60E-03 |
| 29 | Left Insula | 3,99E-03 | 6,64E-05 |
| 30 | Right Insula | 6,17E-02 | 6,86E-03 |
| 31 | Left Anterior Cingulum | 0 | 0 |
| 32 | Right Anterior Cingulum | 0 | 0 |
| 33 | Left Middle Cingulum | 1,23E-02 | 8,40E-04 |
| 34 | Right Middle Cingulum | 7,98E-04 | 1,20E-05 |
| 35 | Left Cingulum | 4,34E-02 | 3,96E-03 |
| 36 | Right Cingulum | 4,08E-02 | 1,15E-03 |
| 37 | Left Hippocampus | **1,43E-01** | 2,16E-02 |
| 38 | Right Hippocampus | **2,25E-01** | 3,97E-02 |
| 39 | Left Parahippocampus | 5,96E-02 | 1,62E-03 |
| 40 | Right Parahippocampus | **1,17E-01** | 1,10E-02 |
| 41 | Left Amygdala | 6,48E-02 | 1,84E-03 |
| 42 | Right Amygdala | 1,03E-02 | 4,54E-04 |
| 43 | Left Calcarine | 7,78E-02 | 1,52E-02 |
| 44 | Right Calcarine | 4,65E-02 | 3,93E-03 |
| 45 | Left Cuneus | 4,14E-02 | 1,38E-03 |
| 46 | Right Cuneus | 3,12E-02 | 4,97E-04 |
| 47 | Left Lingual | 1,03E-02 | 3,10E-04 |
| 48 | Right Lingual | 9,21E-03 | 5,08E-04 |
| 49 | Left Superior Occipital | **1,39E-01** | 8,83E-03 |
| 50 | Right Superior Occipital | **1,18E-01** | 7,87E-03 |
| 51 | Left Middle Occipital | 4,07E-02 | 2,16E-03 |
| 52 | Right Middle Occipital | 7,65E-02 | 3,94E-03 |
| 53 | Left Inferior Occipital | 0 | 0 |
| 54 | Right Inferior Occipital | 8,79E-04 | 8,79E-06 |
| 55 | Left Fusiform | 2,03E-02 | 2,98E-04 |
| 56 | Right Fusiform | 3,40E-01 | 6,58E-02 |
| 57 | Left Postcentral | 0 | 0 |
| 58 | Right Postcentral | 6,50E-03 | 8,87E-05 |
| 59 | Left Superior Parietal | 4,49E-02 | 2,14E-03 |
| 60 | Right Superior Parietal | 7,61E-02 | 4,36E-03 |
| 61 | Left Inferior Parietal | 1,28E-02 | 2,70E-04 |
| 62 | Right Inferior Parietal | 1,41E-02 | 2,58E-04 |
| 63 | Left Supramarginal | 1,74E-02 | 2,86E-04 |
| 64 | Right Supramarginal | 8,86E-03 | 1,56E-04 |
| 65 | Left Angular | 7,68E-04 | 7,68E-06 |
| 66 | Right Angular | 4,39E-02 | 2,30E-03 |
| 67 | Left Precuneus | 1,59E-01 | 1,58E-02 |
| 68 | Right Precuneus | 7,09E-02 | 6,82E-03 |
| 69 | Left Central Paracentral Lobule | 0 | 0 |
| 70 | Right Central Paracentral Lobule | 0 | 0 |
| 71 | Left Caudate | **6,03E-01** | **3,99E-01** |
| 72 | Right Caudate | **7,50E-01** | **6,04E-01** |
| 73 | Left Putamen | 0 | 0 |
| 74 | Right Putamen | 2,86E-03 | 4,77E-05 |
| 75 | Left Pallidum | 0 | 0 |
| 76 | Right Pallidum | 0 | 0 |
| 77 | Left Thalamus | **1,49E-01** | 7,09E-02 |
| 78 | Right Thalamus | **1,89E-01** | 6,16E-02 |
| 79 | Left Heschl | 0 | 0 |
| 80 | Right Heschl | 0 | 0 |
| 81 | Left Superior Temporal | 1,96E-02 | 7,83E-04 |
| 82 | Right Superior Temporal | 4,93E-03 | 1,27E-04 |
| 83 | Left Superior Temporal Pole | 5,55E-02 | 3,92E-03 |
| 84 | Right Superior Temporal Pole | 2,08E-03 | 3,28E-05 |
| 85 | Left Middle Temporal | 7,12E-03 | 4,07E-04 |
| 86 | Right Middle Temporal | 2,20E-02 | 4,87E-04 |
| 87 | Left Middle Temporal Pole | 7,08E-03 | 3,12E-04 |
| 88 | Right Middle Temporal Pole | 2,18E-01 | 1,53E-02 |
| 89 | Left Inferior Temporal | 9,15E-04 | 1,07E-05 |
| 90 | Right Inferior Temporal | 1,63E-01 | 1,64E-02 |
| 91 | Left Cerebellum | 1,99E-04 | 1,99E-06 |
| 92 | Right Cerebellum | 0 | 0 |
| 93 | Left Cerebellum 3 | 0 | 0 |
| 94 | Right Cerebellum 3 | 0 | 0 |
| 95 | Left Cerebellum 4-5 | 0 | 0 |
| 96 | Right Cerebellum 4-5 | 0 | 0 |
| 97 | Left Cerebellum 6 | 0 | 0 |
| 98 | Right Cerebellum 6 | 8,68E-04 | 2,89E-05 |
| 99 | Left Cerebellum 7b | 0 | 0 |
| 100 | Right Cerebellum 7b | 0 | 0 |
| 101 | Left Cerebellum 8 | 0 | 0 |
| 102 | Right Cerebellum 8 | 7,14E-03 | 1,63E-04 |
| 103 | Left Cerebellum 9 | 0 | 0 |
| 104 | Right Cerebellum 9 | 1,09E-03 | 1,09E-05 |
| 105 | Left Cerebellum 10 | 0 | 0 |
| 106 | Right Cerebellum 10 | 0 | 0 |
| 107 | Left Vermis 1-2 | 0 | 0 |
| 108 | Right Vermis 1-2 | 0 | 0 |
| 109 | Left Vermis 3 | 0 | 0 |
| 110 | Right Vermis 3 | 0 | 0 |
| 111 | Left Vermis 4-5 | 0 | 0 |
| 112 | Right Vermis 4-5 | 0 | 0 |
| 113 | Left Vermis 6 | 0 | 0 |
| 114 | Right Vermis 6 | 0 | 0 |
| 115 | Left Vermis | 0 | 0 |
| 116 | Right Vermis | 0 | 0 |
| 117 | Left Vermis 8 | 0 | 0 |
| 118 | Right Vermis 8 | 4,48E-03 | 4,48E-05 |
| 119 | Left Vermis 9 | 0 | 0 |
| 120 | Right Vermis 9 | 0 | 0 |
| 121 | Left Vermis 10 | 0 | 0 |
| 122 | Right Vermis 10 | 0 | 0 |
