# Supplementary material for: Prediction of amyloid pathology in cognitively unimpaired individuals using voxel-wise analysis of longitudinal structural brain MRI
Source: Alzheimers Res Ther. 2019 Aug 17;11:72. doi: 10.1186/s13195-019-0526-8 (PMC6698344; doi:10.1186/s13195-019-0526-8)
Supplement: Supplementary file 2 — Table S2. List of cognitively unimpaired subjects with follow-up times in the interval 3.5 > Δt > 2.5 years used in the analysis. We report their ADNI ID, the research facility and type of scanner. (DOCX 90 kb) [file 13195_2019_526_MOESM2_ESM.docx]

**Table S2.** List of cognitively unimpaired subjects with follow-up times in the interval 3.5 > Δt > 2.5 years used in the analysis. We report their ADNI ID, the research facility and type of scanner.

| **RID** | **DX** | **SITEID** | **FIELD_STRENGTH** |
| --- | --- | --- | --- |
| 96 | Control | 10/16 (m60,m72) | 1.5T |
| 118 | Control | 13/19(m60,m72) | 1.5T |
| 260 | Control | 114/47 (m60,m72, m84, 96, 108) | 1.5T |
| 352 | Control | 45/39 (m60,m72, m84, 96) | 1.5T |
| 498 | Control | 103/4 (m48,m60,m72) | 1.5T |
| 519 | Control | 110/11 (m48,m60,m72) | 1.5T |
| 559 | Control | 101 | 1.5T |
| 619 | Control | 101 | 1.5T |
| 637 | Control | 108 | 1.5T |
| 672 | Control | 17/25(m48) | 1.5T |
| 677 | Control | 16/22(m60,m72,m84) | 1.5T |
| 685 | Control | 101 | 1.5T |
| 923 | Control | 52/23(m48,m60,m72,m84,m96) | 1.5T |
| 1169 | Control | 16/22(m60,m72,m84) | 1.5T |
| 1250 | Control | 57/29(m48,m72,m84) | 1.5T |
| 257 | PreAD | 32 | 1.5T |
| 259 | PreAD | 114/47 (m60,m72, m84, 96) | 1.5T |
| 295 | PreAD | 101 | 1.5T |
| 479 | PreAD | 16/2 (m60,m72) | 1.5T |
| 403 | PreAD | 13 | 1.5T |
| 520 | PreAD | 110/11 (m48,m60,m72) | 1.5T |
| 555 | PreAD | 6/24 (m48,m60,m72, m96) | 1.5T |
| 984 | PreAD | 9/15(m48,m60,m72,m84,m96) | 1.5T |
| 1098 | PreAD | 52/23(m48,m60,m72,m84,m96,m120) | 1.5T |
| 1276 | PreAD | 109 | 1.5T |
