# Supplementary material for: Prediction of amyloid pathology in cognitively unimpaired individuals using voxel-wise analysis of longitudinal structural brain MRI
Source: Alzheimers Res Ther. 2019 Aug 17;11:72. doi: 10.1186/s13195-019-0526-8 (PMC6698344; doi:10.1186/s13195-019-0526-8)
Supplement: Supplementary file 3 — Table S3. Performance of the system using a different feature selection method (l1-norm selection) and evaluated on the interval 3.5 > Δt > 2.5 years. The number of features depends on the l1-norm regularization parameter and on the training data. We report the average number of features used periteration of the evaluation loop. (DOCX 14 kb) [file 13195_2019_526_MOESM3_ESM.docx]

| Table S3. Performance of the system using a different feature selection method (l1-norm selection) and evaluated on the interval 3.5 > Δt > 2.5  years. The number of features depend on the l1-norm regularization parameter and on the training data. We report the average number of features used per iteration of the evaluation loop. | | | | | | | | | |
| --- | --- | --- | --- | --- | --- | --- | --- | --- | --- |
|  |  |  |  |  |  |  |  |  |  |
|  |  |  |  |  |  |  |  |  |  |
|  |  |  |  |  |  |  |  |  |  |
|  | | |  |  |  |  |  |  |  |
| **Regularization parameter  (mean #features)** | **AUC** | **Balanced accuracy** | | | **Accuracy** | **Precision** | **Sensitivity** | **Specificity** | **Fscore** |
| **20000 (205.31)** | 0,748 | **0,651** | | | **0,575** | **0,303** | 0,777 | **0,525** | **0,429** |
| **50000 (576.17)** | 0,742 | 0,628 | | | 0,534 | 0,277 | 0,783 | 0,472 | 0,4046 |
| **100000 (1286.95)** | **0,8** | 0,634 | | | 0,537 | 0,28 | 0,8 | 0,4725 | 0,41 |
| **500000 (3814.73)** | 0,768 | 0,64875 | | | 0,541 | 0,286 | 0,817 | 0,48 | 0,42 |
| **1000000 (5130.59)** | 0,77 | 0,65 | | | 0,54 | 0,283 | **0,823** | 0,47 | 0,42 |
