# Supplementary material for: Prediction of amyloid pathology in cognitively unimpaired individuals using voxel-wise analysis of longitudinal structural brain MRI
Source: Alzheimers Res Ther. 2019 Aug 17;11:72. doi: 10.1186/s13195-019-0526-8 (PMC6698344; doi:10.1186/s13195-019-0526-8)
Supplement: Supplementary file 4 — Table S4. Performance of the system trained on the interval 3.5 > Δt > 2.5 years and evaluated in all other cases. (DOCX 15 kb) [file 13195_2019_526_MOESM4_ESM.docx]

| Table S4. Performance of the system trained on the interval 3.5 > Δt > 2.5 years and evaluated in all other cases. | | | | | | | |
| --- | --- | --- | --- | --- | --- | --- | --- |
|  |  |  |  |  |  |  |  |
| **#features (%)** | **AUC** | **Balanced accuracy** | **Accuracy** | **Sensitivity** | **Precision** | **Specificity** | **Fscore** |
| **6 (0.001)** | 0,57 | 0,52 | 0,44 | 0,66 | 0,21 | 0,39 | 0,31 |
| **65 (0.01)** | 0,59 | 0,54 | 0,46 | 0,68 | 0,23 | 0,4 | 0,33 |
| **653 (0.1)** | 0,63 | 0,56 | 0,5 | 0,66 | 0,24 | 0,46 | 0,34 |
| **1633 (0.25)** | 0,62 | 0,55 | **0,52** | 0,61 | 0,24 | **0,49** | 0,33 |
| **3266 (0.5)** | 0,62 | 0,55 | **0,52** | 0,59 | 0,24 | 0,5 | 0,33 |
| **6532 (1)** | 0,62 | 0,55 | 0,51 | 0,61 | 0,24 | **0,49** | 0,33 |
| **13064 (2)** | 0,64 | 0,56 | **0,52** | 0,63 | 0,24 | **0,49** | 0,34 |
| **32661 (5)** | **0,65** | 0,58 | 0,5 | 0,71 | 0,25 | 0,44 | 0,36 |
| **65323 (10)** | 0,64 | **0,59** | 0,51 | **0,73** | **0,26** | 0,45 | **0,37** |
|  |  |  |  |  |  |  |  |
